# Chronic Recurrent Multifocal Osteomyelitis and Its Management

**DOI:** 10.7759/cureus.18872

**Published:** 2021-10-18

**Authors:** Amman Yousaf, Shoaib Muhammad, Sohaib Bassam Mahmoud Zoghoul, Syed Intekhab Alam, Ahmed Mounir Elsyaed

**Affiliations:** 1 Internal Medicine, McLaren Flint, Michigan State University, Flint, USA; 2 Radiology, Services Institute of Medical Sciences, Lahore, PAK; 3 Urology, Ghulab Devi Hospital, Al-Aleem Medical College, Lahore, PAK; 4 Diagnostic and Interventional Radiology, Hamad Medical Corporation, Doha, QAT; 5 Musculoskeletal Radiology, Hamad Medical Corporation, Doha, QAT; 6 Orthopaedics Surgery, Hamad Medical Corporation, Doha, QAT; 7 Orthopaedics Surgery, Weill Cornell School of Medicine, Doha, QAT

**Keywords:** recurrent osteomyelitis, chronic recurrent multifocal osteomyelitis, chronic osteomyelitis, sapho, crmo

## Abstract

Chronic recurrent multifocal osteomyelitis (CRMO) is an inflammatory disorder of bones first reported by Giedion et al. in 1972. It is a disease of childhood, comparable to SAPHO (synovitis, acne, pustulosis, hyperostosis, and osteitis) in adults. CRMO presents with pain and swelling overlying the involved bones. Inflammatory markers are usually raised and X-rays usually show sclerotic lesions. MRI demonstrates the extent of the lesions accurately and associated soft tissue changes. Nonsteroidal anti-inflammatory drugs (NSAIDs) and corticosteroids are the mainstays of the management. We report three patients who presented with bone pains. Extensive workup and radiological modalities along with clinical findings supported the diagnosis of CRMO. This article highlights important clinical presentations, radiological findings, and various management options.

## Introduction

Chronic recurrent multifocal osteomyelitis (CRMO) is an auto-inflammatory disorder of bones. Giedion et al. first reported CRMO in 1972 [1]. It is typically seen in the young age group and is related to innate immunity. Chronic nonbacterial osteomyelitis is another name of CRMO. Typical clinical symptoms include pain, swelling, and local skin changes. Multiple bones involvement has been reported. Being a rare entity, there are no set criteria for the diagnosis. Therefore, it is a diagnosis of exclusion. Synovitis, acne, pustulosis, hyperostosis, and osteitis (SAPHO) syndrome shows similar findings with CRMO but presents in the adult age group [2]. This article includes clinical presentation, workup, and management for three patients who presented with symptoms of CRMO. Our study also highlights the response to the various treatment options.

## Case presentation

Case 1

**Figure 1:**
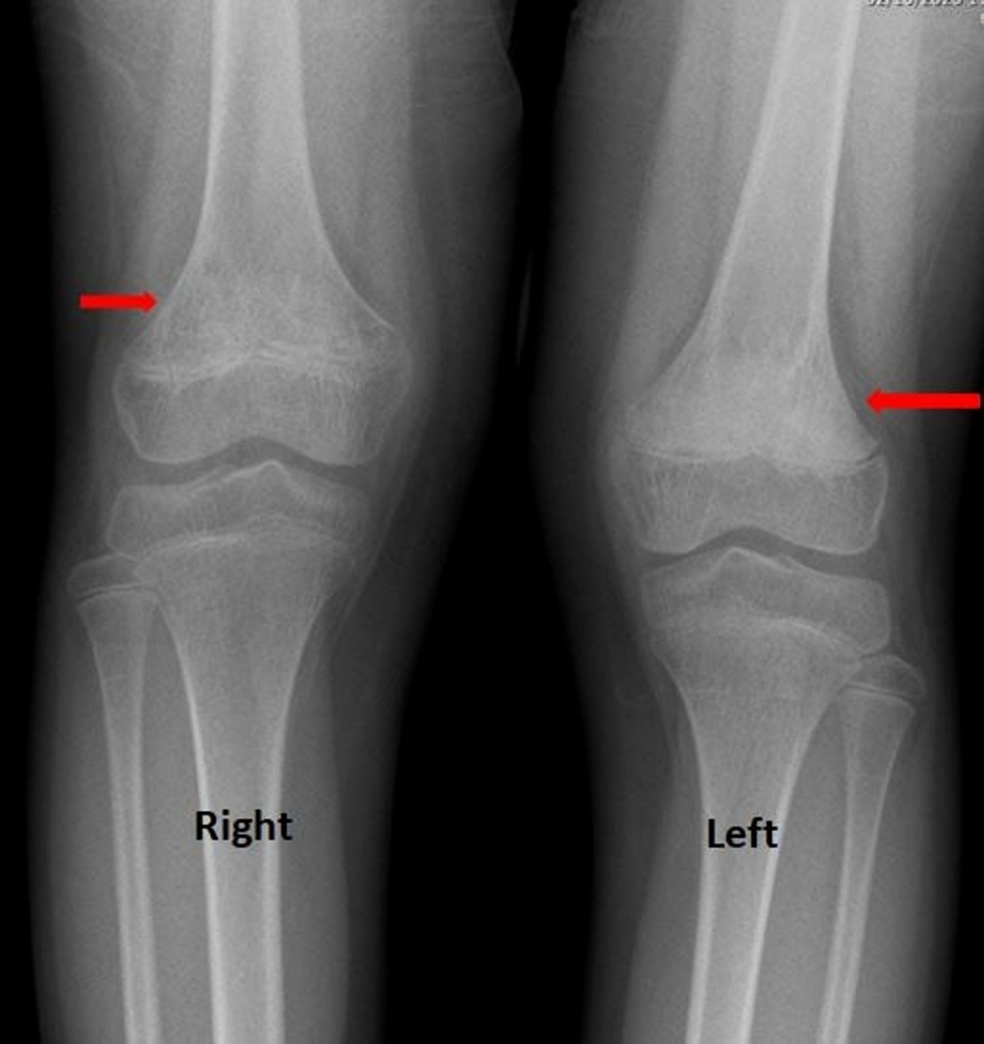
X-ray of both knees. It shows sclerosis in the distal femoral metaphysis, more severe on the left side (long red arrow) as compared to the right (short red arrow).

An 11-year-old child presented to the emergency department (ED) with worsening left knee pain for the last three days. The pain had no identifiable etiology and the patient did not have fever, anorexia, or weight loss. Past medical history revealed a similar episode following minor trauma around three years ago which resolved spontaneously. On examination, there was no tenderness, warmth, or swelling. However, there was slight limping of both lower limbs. The active and passive ranges of motion on the knee and hip joints were within the normal limits. X-ray of knees showed sclerosis of the left femoral metaphysis and to a lesser extent of the right femoral metaphysis as well (Figure 1).

**Figure 2:**
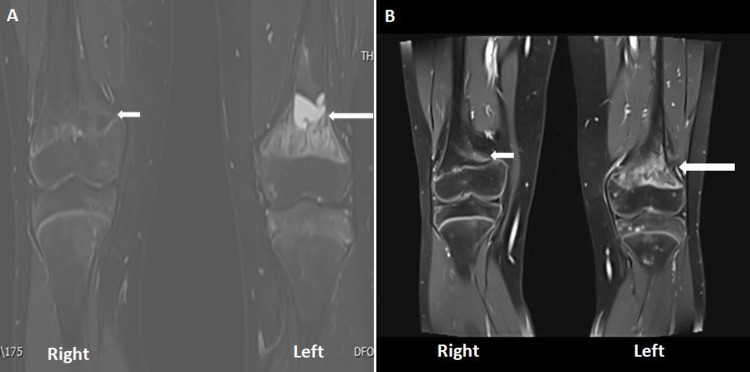
Gadolinium-enhanced MRI of the knee. A: Selected STIR sequence: it shows high signal intensity lesions in the distal metaphysics of both femurs, more severely involving the left side.  B: Gadolinium-enhanced sequence demonstrating heterogeneous intense enhancement on the left side in the affected metaphyseal region of the left knee (long white arrow) and mild enhancement on the right side (short white arrow). STIR, short tau inversion recovery

MRI demonstrated abnormal marrow signals in the both distal femoral metaphysis and in the periphyseal region (Figure 2A).

The lesion was more extensive with cystic change at the left distal femoral metaphysis, and there was a heterogeneous enhancement on post-contrast images (Figure 2B). These findings were concerning for the underlying malignant process. However, the biopsy confirmed the absence of atypical or malignant cells and the culture was negative. Taking into consideration the clinical, radiological, and histopathological features of these lesions the diagnosis of CRMO was concluded.

The patient was managed with oral naproxen, omeprazole, prednisone, and a single dose of 30 mg pamidronate. On his follow-up after a month, the pain improved significantly. On a three months follow-up X-ray of both knees revealed resolution of the sclerotic lesions and the patient's symptoms did not relapse till date.

Case 2

**Figure 3:**
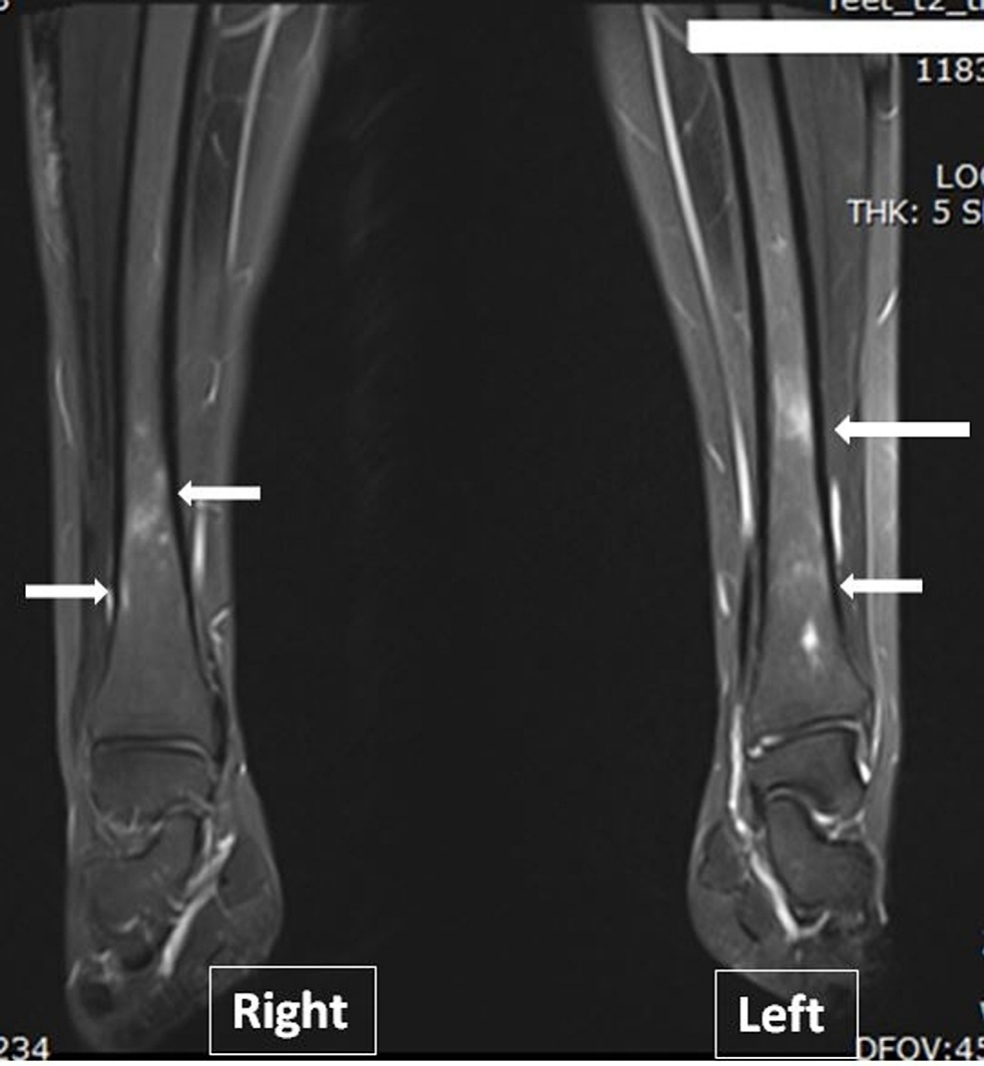
Selected STIR sequence of MRI both legs. It shows high signal intensity multiple lesions involving both tibias. STIR, short tau inversion recovery

A 15-year-old female presented for the radiological investigation to assess the response to her treatment. She was diagnosed with CRMO at the age of 13 with pain in the right and then bilateral wrists and back. Her initial X-ray was unremarkable and MRI revealed multiple patchy abnormal bone marrow signals involving the distal metaphysis at the distal end of the tibia, distal metaphysis of the fibula, the middle part of the talus bone, most of the calcaneal and navicular bone, and proximal part of the first metatarsal bone (Figure 3).

Investigations including biopsy and genetic testing excluded malignancy and genetic syndromes. She was instituted on naproxen for effective analgesia. A repeated MRI three months later showed persistent active disease that warranted the institution of pamidronate. Follow-up imaging due to elevated cardiopulmonary resuscitation (CPR) and erythrocyte sedimentation rate (ESR) revealed the absence of any improvement that confirmed the inefficacy of pamidronate. The patient was then started on canakinumab (interleukin-1 blocker) 150 mg subcutaneously every eight weeks. Despite decreasing dose interval, the disease showed no improvement on imaging and she continued to have elevated acute phase reactants. Ultimately, infliximab 5 mg/kg every eight weeks and methotrexate 10 mg weekly instituted. Fortunately, she showed excellent clinical response and improvement in the laboratory and radiological investigations and has had no relapse of the disease till date.

Case 3

**Figure 4:**
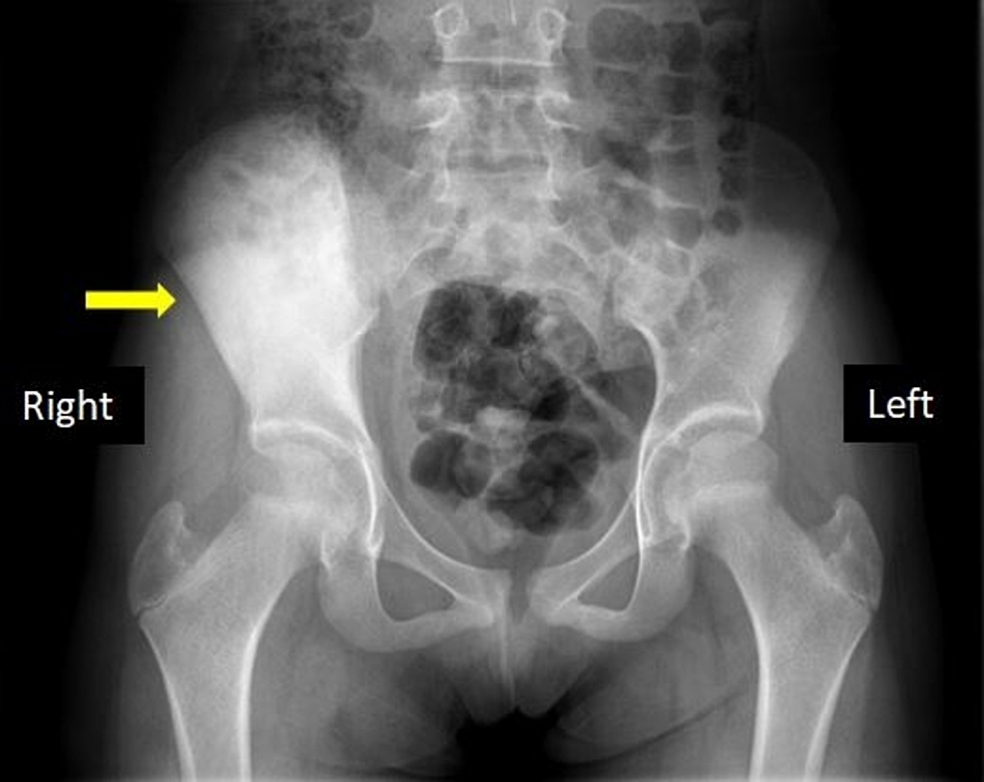
X-ray pelvis. It shows diffusely sclerotic right hip bone (yellow arrow).

An 11-year-old girl presented to the emergency department (ED) with intermittent right hip pain for the last two weeks. Her pain showed exacerbation during the night and worsened gradually with time. Physical examination was unremarkable; however, the range of motion was reduced. Laboratory investigation was significant for increased C-reactive protein (CRP). X-ray showed enlarged and sclerotic bilateral iliac bones more on the right side (Figure 4).

**Figure 5:**
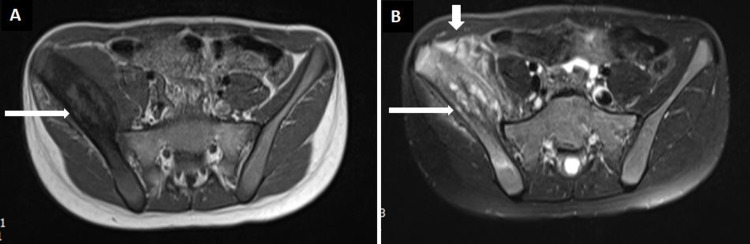
MRI of pelvis. A: T1-Weighted axial section demonstrating right iliac bone and surrounding soft tissue expansion and abnormal signals. B: T2-Weighted axial section at the same level showing heterogeneous high signals in the right iliac bone and surrounding soft tissue expansion and edema.

On MRI contrast, there was heterogeneous intensity expansion of the right iliac bone (Figure 5A). T2-weighted images showed non-homogeneous high intensity lesions in the affected areas (Figure 5B).

**Figure 6:**
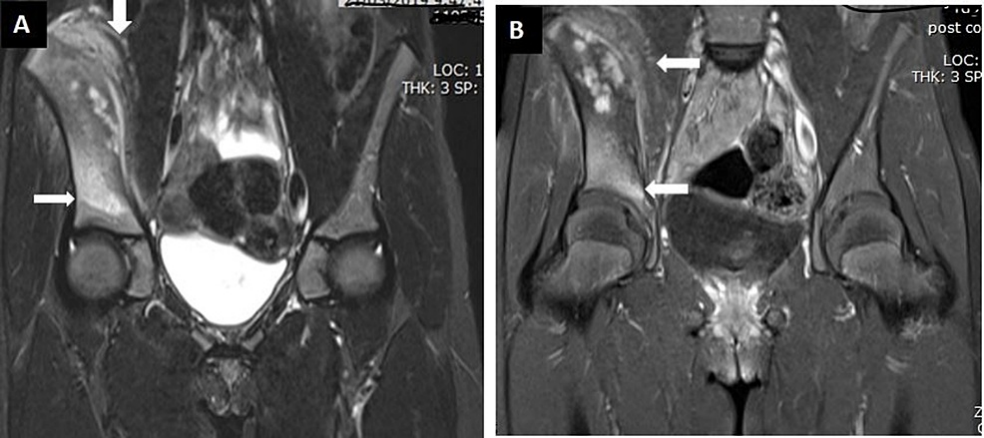
MRI of the pelvis (coronal sections). A: STIR sequence showing abnormal signal enhancement in the right ilium extending to the acetabular roof (horizontal white arrow). It also displays abnormal signal enhancement in the right iliacus muscle (vertical white arrow). B: T1 post-contrast sequence displaying enhancement in the affected areas in the right ilium (white arrow). STIR, short tau inversion recovery

The adjacent part of the iliacus and gluteal muscles showed altered intensity in the pre-contrast sections (Figure 6A), while increased enhancement was seen in the postcontrast sections (Figure 6B). The mass could be seen extending to the acetabular roof.

These findings raised the suspicion Ewing sarcoma on top of the differentials. Biopsy from the right iliac bone showed multiple fragments of cortical bone as well as cancellous bony fragments. There was reactive new bone formation with numerous osteoblasts and associated with loose fibrous stroma in between and aggregation of chronic inflammatory cells particularly plasma cells. There was no evidence of granulomas or malignancy. Therefore, she was instituted on NSAIDs and paracetamol with continuous surveillance. MRI was done after nine months which showed improved right iliac lesions concomitant with her clinical improvement.

## Discussion

Chronic recurrent multifocal osteomyelitis is an autoinflammatory disease and is also known as chronic non-bacterial osteomyelitis. This usually presents in children with a mean age of 10 years. Due to the disease being rare, there is a significant delay in the diagnosis. Similar to our article, female predominance is noted in the literature. Interleukin-6, interleukin-10, and tissue necrosis factor-a are thought to play a part in the development of the disease [3]. A recent study has noted genetic contribution to CRMO with deficiency of interleukin-1 receptor antagonist (DIRA) due to mutation in IL1RN (interleukin-1 receptor antagonist gene) [4]. However, the exact pathophysiology is still not known. A few of the patients also present with other autoinflammatory disorders like inflammatory bowel disease [5].

Chronic recurrent multifocal osteomyelitis presents as an asymptomatic disease with a wide variety of symptoms with pain being the most common symptom [6]. Typically, patients present with dull pain and swelling on the site of bone involvement. Local skin changes include tenderness and redness. Similarly, pain was the main presenting complaint in all our patients. Usually, involved locations include long bone metaphysis and vertebral bodies [7]. Two of our cases had long bones involvement, whereas one patient had a lesion in the iliac bone that is quite rare. Laboratory investigations typically show raised inflammatory markers like ESR, CRP, and leukocytosis analogous to our cases.

X-rays are the primary imaging modality and show lesions to be osteolytic in nature with surrounding sclerosis. The imaging modality of choice is MRI and it shows sites and extent of disease. Few of the important findings of MRI include soft tissue and bone marrow edema, periostitis, and extension across the physis [8]. All of our patients were assessed with MRI to know the extent and severity of the disease and to check the response to the medication. As CRMO is a diagnosis of exclusion, laboratory and radiological investigations are pivotal to exclude infection and malignancy in order to favor the diagnosis of CRMO. There are no universal diagnostic criteria and Bristol diagnostic criteria developed by Roderick et al. can be used to reach the diagnosis. To confirm the exclusion of malignancy, a bone biopsy is usually done.

Multiple studies have demonstrated the role of NSAIDs as first-line therapy in the treatment of CRMO [9]. This was also seen in our patients. Corticosteroids show rapid improvement similar to our patient but have a setback of rarely causing remission in the long run. If the patient responds to corticosteroids but disease flares come up, high dose corticosteroids can be applied. Due to pathophysiology related to IL-1, various studies have shown the efficacy of IL-1 blockers including canakinumab [10]. However, in our patient, treatment with canakinumab failed and other medications were used to control the disease progression. In patients who fail therapy with NSAIDs and corticosteroids, disease-modifying antirheumatic drugs (DMARDs) are used. These include TNF-α inhibitors, sulfasalazine, or methotrexate [11]. One of our patients failed therapy with NSAIDs, corticosteroids, and IL-1 blockers (canakinumab). Ultimately, she was started on infliximab (monoclonal TNF-a antibody) and methotrexate (DMARDs) and showed an excellent response.

## Conclusions

Chronic recurrent multifocal osteomyelitis is an auto-inflammatory disorder of bones and it presents with soft tissue swellings and bone pain. MRI is an excellent modality to delineate the extent of disease and it can also demonstrate the response to treatment. From our cases and review of the literature, we conclude that CRMO is a diagnosis of exclusion and biopsy should be performed to surely rule out the underlying malignant processes. Most patients respond well to NSAIDs and corticosteroids; however, interleukin 1 inhibitors and the role of DMARDs role have also been illustrated in the literature.
